# Using longitudinal qualitative research to explore extra care housing

**DOI:** 10.1080/17482631.2019.1593038

**Published:** 2019-04-02

**Authors:** Ailsa Cameron, Eleanor K. Johnson, Liz Lloyd, Simon Evans, Randall Smith, Jeremy Porteus, Robin Darton, Teresa Atkinson

**Affiliations:** a School for Policy Studies, University of Bristol, Bristol, UK; b Association for Dementia Studies, University of Worcester, Institute of Health and Society, Worcester, UK; c Housing Learning and Improvement Network, London, UK; d Personal Social Services Research Unit, University of Kent, Kent, UK

**Keywords:** Qualitative longitudinal research, older people, extra care housing, housing with care, care needs

## Abstract

**Purpose**: The process of individual ageing in the context of a care environment is marked by continuity and change. It is shaped by individual, health-related factors as well as by diverse social and environmental factors, including characteristics of the places where older people live. The aim of this paper was to explore how longitudinal qualitative research, as a research method, could be used to explore older people’s changing care needs.

**Methods**: The study used a longitudinal design to examine how the care and support needs of residents and their expectations of services developed over time and how these were influenced by changes in the organisation of their housing as well as in the make-up of the resident population. Residents were interviewed on four occasions over 20 months.

**Results**: The study highlighted the complex ways in which some participants proactively managed the care and support they received, which we argue would have been difficult to discern through other methods.

**Conclusion**: The study adds to the growing evidence base that supports the use of qualitative longitudinal research; the approach enables the researcher to capture the diverse and mutable nature of older people’s experiences at a time of profound change in their lives.

## Introduction

In the UK, the increase in life expectancy is frequently associated with long-term health conditions, disabilities and a loss of ability to manage self-care, which have led to increased demand for health and social care services (Atkinson et al., 2014; King’s Fund, 2014). Extra care housing (ECH) is a form of housing with care built specifically for older people, often known as “assisted living” or “senior housing” in the USA, Canada and Australia (Atkinson et al., 2014). ECH has a particular place in the range of social care services. It offers a distinct model which facilitates independent living for older adults with access to care and support when necessary. There is therefore a social and temporal dimension to the study discussed in this paper, with attention to the lifecourse experiences of older residents, their perceptions of their health and wellbeing as it changes over time, their experiences of living in ECH, and how these interacted and influenced their perceptions of ageing and receiving care services.

### The context of ECH

The concept of the lifecourse focuses attention on time, change and continuity within individual lives and the interrelationship of individual lives and social contexts (Dannefer & Stettersten, 2010; Haraven & Adams, 1982). Individual experiences are shaped by historical period as well as by social and economic inequalities. Moving into an ECH “scheme” (or facility) is a major event in an individual’s life but this event also needs to be understood in the wider social context of housing and care. ECH represents a particular contemporary perception of the optimal conditions for living when in need of care in old age (Shaw, West, Hagger, & Holland, 2016). At the same time, it is undergoing significant changes as a result of contemporary political and economic pressures faced in England (Yeandle, 2016). For example, there is an increase in the number of schemes for older people who wish to purchase a place, but a shrinking number for those who rely on state support to rent (LaingBuisson, 2016) giving rise to concerns among providers and commissioners about the future of ECH for economically disadvantaged older people.

ECH offers those who need support the possibility of obtaining this in a way that maximizes their capacity for independent living. It thus reflects the paramount cultural values of independence and choice, which are ubiquitous in social policies although severely tested in practice by cuts in public expenditure (Bligh, Cairncross, & Porteus, 2015). Unlike the more communal arrangements of a care home, residents of ECH schemes have their own apartment behind their own front door, which signifies their control over the space they occupy, how they wish to live and who comes and goes. At the same time, care is provided as and when it is needed so that residents can avoid the pitfalls of “staying put,” such as social isolation and “inappropriate housing” (Callaghan & Towers, 2014, p. 1429). Care needs are assessed prior to admission and/or when new needs appear to have emerged. Usually, each individual resident has a plan for care and support provided routinely. In addition, a member of staff is available 24 hours a day, 7 days a week, should unexpected needs arise (Atkinson et al., 2014; Darton et al., 2012). “Care” typically includes personal care, for example help with washing and dressing or taking medication, while “support” refers to help with domestic chores, such as cleaning, shopping or social activities within the schemes or in local communities (Evans et al., 2017). Care and support is typically provided by an on-site care team or, occasionally in keeping with the personalisation agenda, by an external provider if arranged by the resident themselves or by their family members.

### Balancing care needs

An important question facing the providers and commissioners of ECH concerns the combination of individual residents with diverse care needs and how particular combinations shape the overall character of a scheme and its ability to function as a community. Managers of individual schemes generally seek a balance of different levels of dependency among residents: those with no or minimal care needs; those with medium-level care needs and those with high-level care needs (Baker, 2002; Wright et al., 2010). The widespread assumption underpinning this approach is that growing dependency will occur over the course of a person’s residency and that, as they age in place at the end of life or move into different settings, new residents with no or minimum care needs will move in. In theory, through this approach, the composition of schemes would be in a state of constant change in terms of the individual residents while maintaining continuity in terms of the overall profile. Not surprisingly, however, such a balance is difficult to achieve in practice (Baker, 2002; Wright et al., 2010). Extended life expectancy at the oldest ages has tended to reduce the death rates of residents and increase the number with high-level care needs, while pressure on public resources has meant that people with high-level care needs are given priority access to publicly-funded schemes over those with lower-level needs. An important element of this research was to investigate how such changes played out in the lives of individual residents and influenced their experiences of ageing and receiving care.

As a way of enabling older people to adapt to their changing health and capacity for self-care, ECH appears to offer a flexible and supportive option, an ideal compromise between the struggle to cope alone and the dread of traditional residential care. Yet, questions arise about how its implied flexibility actually works in practice, how responsive different schemes are to the changing needs of individuals over time and how ECH can retain its ideal as a way of maximizing older people’s independence and control when its residents have more complex needs as a result of ill health and disability.

### Contribution

Using a longitudinal qualitative research (LQR) approach, the aim of The Provision of Social Care in Extra Care Housing (ECHO) project was to investigate how care is negotiated and delivered in ECH. Focusing on the “extra care” element of extra care housing services, the ECHO project explored the perspectives of residents on their changing care needs and their experiences of being cared for. Data were gathered through a range of methods in four “rounds” over the course of one and a half years. The aim of this paper is to explore how longitudinal qualitative research, as a research method, could be used to explore older people’s changing care needs. We reflect on the value of the LQR approach for exploring continuity and change at the individual and organisational levels as well as considering its particular strengths in enabling us to identify the interconnections between these different levels. This paper focuses particularly on the perspectives of the residents and draws on data gathered from them directly. The findings contribute to a better understanding of ageing and care as well as providing useful information about the potential of care provision in specialist housing.

### Rationale for the research design

A longitudinal qualitative approach was adopted as the most appropriate for an exploration of processes involved in the giving and receiving of care through a period of individual and organizational change. Longitudinal qualitative research focuses the researcher’s attention on both individual and social levels and the interactions between these (Neale & Flowerdew, 2003). It provides a means of understanding how experiences and processes change over time (Corden & Millar, 2007a) and, as Thomson (2007) suggests, allows the researcher to explore the outcomes of changes. Consequently, LQR has particular resonance for policy researchers, athe “long” view offered by qualitative longitudinal research offers the possibility of developing more complex and thus realistic understandings of how and why individuals and communities live as they do as well as the intended and unintended consequences of the policies themselves (Thomson, 2007, p. 572).

Corden and Millar (2007b) note the importance of lifecourse approaches which address the significance of time and change, especially in periods of transition. The decision to move to ECH is deeply significant in terms of individual ageing and might be regarded as a major transition in the lifecourse (Grenier, 2012). Moreover, our research focused not only on the individual residents but also on the ECH schemes as organizations, whose approach and capacity to care is shaped by broader economic and political contexts. There were, therefore, interconnections between individual experiences and the life of the organization where the research took place.

In the context of ageing and social care, the transitions that participants are living through can be difficult to discuss, especially when participants are in need of personal care and when their ability to manage their daily routines independently has diminished. A major advantage of LQR methods is that interviews carried out over time promote familiarity and trust between participant and researcher and enable a researcher to raise sensitive topics at a more opportune moment, so avoiding the possibility of raising feelings of embarrassment that would be more likely in a one-off interview (Lloyd et al., 2017).

There is no accepted standard concerning the duration of longitudinal research (Corden & Millar, 2007b). What is significant is that there should be an expectation that change will occur over the timeframe selected. For this study, an 18-month period for fieldwork was selected based on previous experience of longitudinal research with older people (Lloyd, et al., 2014), which suggested that changes in care needs would be evident among our sample within this time frame. Additionally, we expected that within this time frame there would be significant changes in the social care policy and practice contexts within which ECH operates and we were confident that we would be able to obtain evidence on the impact of these changes on the schemes in our fieldwork.

## Methods

We recruited four ECH schemes to take part in the study, one of which provided specialist support to people living with dementia. These schemes were based in two areas: a unitary authority (Area 1), and a county council, two-tier authority (Area 2). Each scheme was visited on four occasions, at six-month intervals and data were gathered through semi-structured interviews (with residents, managers, care staff and local commissioners of housing and care); analysis of documents and unstructured observations. The analysis of documents, such as annual reports, provided valuable contextual information about organizational priorities and values. The observational data helped shape questions asked in the interviews with residents, for example in relation to opportunities to take part in social activities. This paper reports data gathered primarily from the interviews with residents.

Our intention was to recruit 10 residents at each site and interview them four times over 18 months. Mindful of the potential for high attrition rates (Mody et al., 2008), we recruited between 12 and 15 residents at each site, see Table 1 for detail. In total, 51 residents took part in the first round of interviews. They ranged in age from 54 to 97 years. There was some diversity in the ages of participants between sites, with the majority of participants at site A being in their 60s while the majority of participants at site D were in their 80s or 90s. In order to reflect the diversity of existing ECH residents, we spoke to both “new” and established residents with the length of time that participants had spent living in ECH at the point of our first interview ranging from one month to 19 years. The majority of participants had lived in ECH for less than two years, reflecting the fact that site C was a newly established ECH site and site D had recently been extended to accommodate more residents. Ten male residents and 41 female residents participated in interviews. While the majority of participants were widowed (n = 22), 14 were divorced or separated, seven were single, and eight were married. All eight participants who were married lived in ECH with their partners. Every resident whom we spoke to disclosed some form of illness or chronic condition. Many had issues with poor mobility and/or arthritis and some had a history of mental health problems, a stroke, cancer, and/or heart problems. Despite this, 19 participants reported that they did not receive any care provision at the time of our first interview. Others received care or support ranging from short “welfare check visits” to four prolonged visits per day. The table below reports how many residents took part in each round of interviews.

**Table 1.** Number of completed and missing resident interviews in each round.

	Round 1		Round 2		Round 3		Round 4		All Rounds	
	Missing	Complete								
	(M)	(C)	M	C	M	C	M	C	M	C
Site A	0	14	4	10	4	10	5	9	13	43
Site B	0	12	3	9	1	11	4	8	8	40
Site C	0	11	9	2	5	6	5	6	19	25
Site D	0	14	0	14	0	14	0	14	0	56
All Sites	0	51	16	35	10	41	14	37	40	164

Interviews in round 1 covered biographical details (including age, relationship status, and the length of time that they had lived in their ECH scheme) as well as residents’ reasons for moving into ECH, their participation in social activities, their social contacts, health status, their care plans, their experiences of care and whether their needs and experiences had changed over time. These interviews lasted between 20 and 75 minutes with the average at approximately 50 minutes. Subsequent interviews (rounds 2–4) explored any changes in their need for or provision of care and the factors that lay behind any changes. These interviews were usually shorter than interviews in round 1, lasting between 21 and 37 minutes. Analysis of data from each round yielded themes that were followed up in subsequent rounds. For example, loneliness and isolation emerged as themes to be followed up and explored in greater depth. Two of the interviewers (AC, EJ) had experience of LQR while the third (TA) had research experience with people living with dementia. After each visit the researchers made brief fieldnotes, for example noting changes in the activities provided for residents. These fieldnotes informed the analysis of interview data as well as the subjects explored during subsequent rounds of interviews. None of the participants was known to the interviewers prior to the start of the research.

All interviews were audio recorded and transcribed in full by a university-approved transcription service or by a member of the research team. Thematic analysis of the transcripts was led and managed by (EJ). A sample of eight transcripts from the first round were read and independently coded by members of the research team using *a priori* codes drawn from the literature as well as thematic codes which emerged inductively from the data. Discussion of these led to the development of the initial coding frame which was used to code the first round of resident interviews using NVivo software. The coding frame was supplemented with additional codes as they emerged inductively both during the course of coding and following each subsequent round of interviews. Analysis of longitudinal qualitative data is a complex process and can be carried out both cross-sectionally, in this case considering all residents’ experiences at a specific moment in time, or longitudinally to understand each individual resident’s experiences over time (Calman, Brunton, & Molassiotis, 2013). Data can also be analysed thematically over time. Our approach combined longitudinal and thematic approaches. For example, we analysed how an individual resident’s needs for care changed over time and specific themes over time as they emerged. These themes included, for example, residents’ perceptions of the resident profile of their schemes and changes in staffing. The themes presented in this paper were chosen to illustrate the contribution that a longitudinal approach can make to our understanding of ECH.

### Ethical issues

The ethical issues faced by researchers undertaking LQR studies are, in the main, the same as those faced in any social research project, although they may be intensified because of the additional demands placed on participants’ time and attention (Corden & Millar, 2007b). In our study, the potential ethical issues were made more complex because of the age of our resident participants and their health conditions throughout the study. Ethical approval was provided by the National Social Care Research Ethics Committee, reference 15/IEC08/0047.

We visited each site to introduce the study before beginning fieldwork. This enabled us to explain the aims and objectives of the study to managers and to secure their personal support for the research. We left information sheets to be circulated to residents and staff. Any resident who wished to participate approached their scheme manager who organized interview dates. We recruited approximately two- thirds of our sample at each scheme through this approach and relied on word of mouth among residents to recruit the remaining participants.

Informed consent, anonymity/confidentiality and safeguarding were all important ethical considerations. All participants were informed about how data would be stored and used anonymously, as well as the limits to confidentiality, and their written consent was given prior to the commencement of each interview. Anonymity and confidentiality are key issues in ensuring the ethical probity of research. Participants’ agreement to take part in the study was based on our agreement to maximize the anonymity of individuals as well as that of the ECH schemes where they lived. Given the potential for participants to reveal that they might be at risk of harm—to themselves, or from a care worker or fellow resident—we offered all participants limited confidentiality and developed a protocol by which members of the team would review and, where appropriate, report any concerns raised. On one occasion, we followed up with a resident an issue that they had spoken about to us and reported to the scheme manager as we wished to make sure that it had been dealt with to their satisfaction.

Asking residents to reflect on changes in their life circumstances can cause distress. To manage this eventuality, we agreed that if a resident became upset during an interview we would change the line of questioning or terminate the interview. Although we never had to end an interview, we did, on occasion, change the line of questioning when we thought participants were becoming upset, usually in response to questions about loneliness but, sometimes, as they reflected on changes in their health.

Although we wanted to include the experiences of people living with dementia in ECH, we decided to include only those who had the capacity to consent to take part in the research. In these cases, our approach was informed by Dewing’s (2008) five-step process consent method. This involved considering how participants with dementia might communicate and express their wishes to engage in the research in distinct ways, such as through verbal and non-verbal indicators and through implied meanings rather than intellectually correct language. The process consent method required that we engaged with managers, care workers and/or residents’ families to establish the usual level of wellbeing of participants with dementia and, in turn, the basis for consent for each individual. We monitored consent in this way on an ongoing basis, both re-establishing the basis for consent on each of our four visits and during interviews if it appeared that a participant’s ability to communicate had become severely reduced. Over the course of the study, we determined that participating in the study was no longer in the best interests of three participants because their dementia had progressed and, in consequence, their ability to communicate how they felt about engaging in the research was severely reduced. These three participants took no further active part in the study. Additionally, seven participants were lost to the study. Three died, two entered nursing homes and two decided to withdraw. A further six participants were unable to take part in all four rounds of interviewing, due to ill-health or hospital stays.

## Results

To illustrate how a longitudinal design informs our understanding of ECH, we present below data addressing our first objective in three key themes, which relate to the experiences of residents: the changing care needs of residents; residents’ perspectives on the mix of residents in ECH schemes where they lived, and residents’ perspectives on organizational changes.

### Changing care needs

Over the course of the study, 13 out of the total sample of 51 residents reported that their care or support needs had changed, such that the total number of hours of care which they received had increased or decreased, either on a temporary or permanent basis. For example, at site D, resident D4 [aged 75] reported at our first visit in January 2016 that having recently recovered from a fall she had stopped receiving the temporary additional care which began following her fall. She said:

I needed help then and they were brilliant then, but I had to go to hospital in the end. … then I came out of hospital and they [care staff] helped me again for a little while but now I’m able to do things for myself but I will need them again because me leg is getting… I’ve had ulcers on my leg and I’m going to need someone to help me with stockings.

During our second and third visit, the same resident told us that she was still managing without any “extra” care. However, by the time of our final visit in the spring of 2017, her health had deteriorated and she was receiving care on a permanent basis to:

put a stocking on for me because I can’t do that myself. I can take it off and you know cream my leg and everything but I can’t get the stocking on. So they come in and do that but that’s the only care I get really.

At site B, resident B2 [aged 97, with heart problems and receiving chemotherapy for lymphoma] told us on our first visit that she didn’t receive any care or support, she said “Whatever I do, I do myself.” By the time of our second visit, however, the picture had changed and she told us:

I used to do everything myself and now I’ve got my niece doing my washing and just tidying up round here for me so I haven’t got that to do and I’ve got a carer come in every night at six o’clock to see if I’m alright but it’s foreign to me you see because I used to do everything myself. I just can’t do it. I just haven’t got the energy.

The idea of having a daily visit from a care worker was suggested to resident B2 by her general practitioner, who suggested that care workers visited her every evening. At this stage, she was also being visited by a district nurse. During our third interview, resident B2 told us that she had “sacked” the care worker after a matter of weeks, resisting the need for a daily visit, and had told the scheme manager “I don’t want them anymore and I didn’t have them anymore.” At the time of our fourth and final visit to site B, this resident had moved into a nursing home following a further decline in health.

A small number of residents at the specialist dementia scheme, site C, also told us about their changing care needs. During our first round of interviews, resident C11 [92 years old] told us “I’m alright at the moment the way I go on. I mean there will come a time when I shall need more care and I shall have to pay more you know, fair enough.” By our fourth visit, resident C11 told us “everything’s changed”, she said:

now the carers have to do the cleaning and I pay for half an hour’s cleaning, £8.00, and that’s been happening about the last three or four weeks, but they’re here for ten minutes … well unless I stand over them, I’m about to grumble about paying for that.

The majority of residents from the four schemes who took part in the study received care and support from the on-site care team but six residents told us that they had chosen to receive all, or part of, their care and support from an alternative provider. While these arrangements remained in place for most residents, a small number of participants reported that they had changed providers over the course of the study. At site A, resident A1 [aged 57, with epilepsy and a history of heart attacks and strokes] received care and support from the on-site team as well as from an independent agency, who cleaned her flat. During her first interview, she told us that she had recently been in hospital and that, on her return, she had had regular visits from the on-site carers who would pop in “to check on me and then they’d ring me and ask me if I was alright on the phone and if I wasn’t well I just had to press the buzzer.” During her third interview, she told us that she continued to have four visits a day from the on-site care team to help her with administering medication but that she had recently decided to discontinue support visits from the external agency, inferring that the service was expensive and deciding that, given her improved health, she would clean her flat herself. She said:

I don’t have my cleaning done no more because they charge £12 for washing and other things. I couldn’t do it before, but I am moving my legs and my back a bit. I know they are going to give me some steroids in my back and give me a course of things to do so I don’t mind doing my own [cleaning]. So I got on with doing things. If I can I do it.

In this sense, taking a longitudinal approach allowed us to demonstrate how some residents decided to manage how their changing care and support needs were responded to and by whom. Residents gave a number of reasons for making changes to the timing, content, and/or provider of their care and support. While, most often, these changes were born out of a change in the nature or degree of a resident’s care needs, other residents described making changes due to the costs of care or support (resident A1), the poor quality of care provided by an external agency (resident A6), or because the on-site care team would not be able to provide the specialized support which was required, such as counselling (resident B4).

### The changing mix of residents

In common with previous research on ECH (Callaghan, Netten, & Darton, 2009; West, Shaw, Hagger, & Holland, 2017), the changing mixture of residents living in ECH was a topic that most participants talked about. Many of the participants at sites A, B and D thought that new residents were moving into ECH with higher and more complex needs than they had done previously. These perspectives were borne out in an interview with a local commissioner of housing with care in sites A and B. This commissioner told us that the local authority had recently decided to change their practice so that, to be eligible for a publicly funded place in ECH, an individual must be in need of a minimum of five hours of care per week. Although this change in practice is not uncommon (see Wright et al., 2010), our LQR approach was able to capture its unintended consequences on the everyday experiences of residents living in ECH.

At our first visit to site B, resident B5 [aged 89 years] told us that she had moved in 12 years ago, at the same time as four other people from the same estate where she had lived, which was due to be demolished. She described how, alongside these four individuals, she had formed a residents’ committee and organized events:

We had holidays away, sort of [name of place] and places. No, it was a very full life when I came here first … but then of course it petered out gradually, because as I said, we’re all getting older and things became more difficult for people.

Also during her first interview, resident B5 described how, although there were still activities organized for residents, fewer people attended these. She attributed this poor attendance partly to the death of her original group of friends but, also, to her perception that there was an increased number of residents at site B who had dementia. She said that these residents “don’t take part … we don’t get any new faces because they seem to want to stay in their flats.” Similar findings have been highlighted in other studies (see West et al., 2017).

The picture had changed a little at the time of our second interview with resident B5, who noted that, although it was “a different set up to when I came,” two new residents had recently moved in, both of whom attended activities:

and they’re a boon because they are a lot younger and you know, sort of with their ideas and that, but that’s good, that’s good, but until then it was the same people all the time.

By our third visit to site B, there were new organized activities, partly at the behest of the new younger residents, with the result that these events were more vibrant and more residents took part, resident B5 noted:

We’ve had some younger people come in, big difference, you know but they seem to join in, more so than the older ones that come in … So yes, we got quite a few groups of things … So there’s something going on most days.

The increase in organized social activities clearly had a positive impact on resident B5’s wellbeing, she noted, “so my time’s really filled. I’m so pleased about it really… Yes, so I’m not moping or anything like that. I got plenty to do and see, so I don’t know where the time goes sometimes.”

So, although all new publicly funded residents at site B were now required to have care needs, some new residents at the scheme were younger than existing residents which, for resident B5, had positively affected the scheme’s community. Adopting a longitudinal approach allowed us to explore how the residents’ relationships and social life within the four sites waxed and waned over time and affected individual residents’ wellbeing.

Participants from across the four sites reflected on their changing relationships with other residents. Some focused on their experiences of moving into ECH and meeting new neighbours. At site D, a new extension had opened shortly before our research began and an influx of newer residents had an impact on social relationships. At the first round, resident D8 [aged 87 and had lived in ECH for eight months] told us he did not know many of the well-established residents, but he had come to know some of those who had recently moved in who were “possibly a bit younger overall and I’m very young in my ways.” At our second visit, resident D8 told us:

It’s always a bit hard work when you first come to a place you don’t know anybody and I think I said last time it’s the new people I’ve got friendly with rather than the people who have been here a long time. They tend to stick to themselves, their little groups when they’re eating and so on and I never manage to merge with any of them when I’ve tried sitting on different tables and didn’t get much of a response, but I’ve done much better with people that moved in at the same time as I did.

By our third visit this resident thought that integration between residents living in the new and older parts of the scheme had improved. He went on to describe that the problems of integration were accentuated by the layout of the building, with the new apartments quite a long way from the main social spaces but he also said “I think there’s partly an age thing in it. Most of the new people are probably a bit younger than the ones who’ve been here a long time, and they’ve got more energy to do things.”

### Organisational changes

The impact of changes in the wider national and local policy contexts was evident in the data we obtained from interviews. For example, residents and staff referred to the higher levels of need of incoming residents, reinforcing evidence from previous studies of ECH and residential care homes (see for example West et al., 2017). Policies in England have tended to emphasize keeping people in their own homes for as long as possible and this has led to higher levels of disability among care home residents. At the same time, there has been a knock-on effect on ECH, which now accepts fewer people who are able to manage with minimal levels of care and support. As our LQR approach was able to highlight, such developments have an impact on the experiences of residents living in ECH.

Organizational changes were evident at all four sites and, not surprisingly, residents reflected upon these changes over the course of the study. The longitudinal nature of the study enabled us to follow participants’ feelings over time, capturing how change was unsettling for many residents. As in previous research (Netten, Darton, Bäumker, & Callaghan, 2011), in three of the sites new managers and care workers were appointed and residents talked about their feelings as they anticipated and lived through these changes. When we first visited site A, the manager had been in post for less than one year and residents felt that she was still finding her feet, with several remarking that she wasn’t very communicative or receptive to suggestions from residents. By the time of our second round of interviews, site A’s manager had left and a new manager had been appointed. Residents were hopeful that this new manager would be more approachable than her predecessor and listen to their complaints, one of which concerned a lack of social activities. Resident A5, for example, said:

I hope the new manager is going to be a bit better than the old one, because the old one wasn’t much with us … But this one seems to be, she come and sat with us a couple of times and I’ve chatted with her. And I hope she’s going to pick up the complaints we got.

During our third visit to site A, resident A5 said “[name of manager] is trying hard to get things going.” The new manager had begun arranging events, such as a Halloween party, and there was talk of a new “activities champion” being appointed. At our final visit, resident A5 said:

When you’ve got three different managers in a short time you’ve got to go with the flow as I say, you know like I’ve got to get used to her and she’s got to get used to us like. She’s different. Totally different but she is doing big changes to the building and she’s brightening things up. I admire her for that.

Changes in management could prove very unsettling for ECH residents, particularly when managers had been in post for a long time and were well regarded. During our third visit to site D we learnt that the manager, who had been in post for several years and was very popular among residents for her friendliness and professionalism, was leaving. Anticipating this change was troubling for many residents. As resident D12 said “We are losing [name] of course, she’s leaving at the end of the year. I shall miss her because with [name] as a manager your problem is her problem.” Resident D1, who had been living in the scheme for just over a year by that stage, was very apprehensive, she said:

I dread her going. It’s very unsettling when some of the main people you know go … It’s just not like just sort of one of the carers or one of the cleaners or someone like that going. But when it’s one of the managers who’s the head of this place … then it can make a lot of difference to a place can’t it?

At our final visit, site D’s new manager was settling in, but residents were finding it hard to get used to her and were inevitably making comparisons between her and her predecessor. For example, resident D5 said “She doesn’t speak to anyone, and we are all complaining … she won’t even say good morning, good afternoon, she’ll walk straight by you.”

At sites A, C, and D, it was changes in management that prompted residents to reflect on the importance of certain managerial practices and styles in promoting positive experiences of ECH. Similar comments were made by residents in relation to care staff at all sites. Our ability to gather this important data with regard to the impact of staff changes on residents’ experiences of ECH was greatly enhanced by the LQR method. A single round of interviews at these schemes would not have yielded such detailed insight into how managerial approaches and organizational changes impact upon residents’ experiences of ECH.

## Discussion

This study differs from previous studies of ECH in that the LQR design enhanced our understanding of how residents’ health and needs for care fluctuated over an 18-month period. Additionally, we were able to explore how their experiences of living in ECH changed during this time, including the impact of changes in the organizational context. The findings of this study reflect the precarious position that older people are in when their need for care and support increases and how practices of care can either exacerbate a sense of precariousness or provide a sense of security (Grenier, Lloyd and Phillipson, 2017). The findings also demonstrate how changes in local ECH eligibility policy were impacting not just on the mix of residents but also on their experience of communal life. Indeed, the findings from this study suggest that contemporary conditions make it harder to promote a sense of security in ECH.

Using a LQR approach allowed us to explore how flexible care and support services were in practice. We were able to demonstrate how changes in need for care and support, on a temporary or permanent basis, were responded to. Across the study, residents were appreciative of ECH’s flexibility, indeed it was often the reason why they chose to enter ECH in the first place. In addition, our LQR approach allowed us to explore the ways in which some residents proactively managed the care and support they received. For example, choosing to stop having additional support to clean their apartment in favour of having more care or making the decision to end their contract with an external agency in favour of using the on-site care team. In this sense, we got a much better appreciation of how care needs are negotiated and responded to within ECH over time.

Like all social settings, ECH schemes are not static entities and our study was able to explore the perceived impact of some organizational changes that happened during our fieldwork. For example, resident interviews explored some of the tensions resulting from the changing nature of the “balance of care” within ECH. Significantly, we were able to explore residents’ perceptions of the impact which new residents, often with higher and more complex needs, had upon the communal life of the schemes.

Over the course of the 18-month study, participants placed an increasing emphasis on how changes in resident mix had impacted upon participation in social activities. Like previous research (Shaw et al., 2016), this study illustrates the significance attached to the social activities organized for residents within ECH settings. It also demonstrates how changes in the frequency of social activities often reflect changes to the organizational context, for example the availability of funding to support activities, the availability of someone to organize events and whether there are residents willing and able to take part in them. Additionally, examining the perspectives of residents longitudinally revealed that relationships between residents in ECH take time to build, as they do in any setting. New residents, with or without care needs, may require time to adjust to their new environments before they engage with social activities. Many residents also experience the loss of friendships as their neighbours move to other settings, become unwell, or die. These changes demonstrate the significance of a lifecourse approach to understanding friendships, both the loss of old friends as well as the advent of new friendships. Taken together, these factors suggest that, over time, the nature of social life within ECH ebbs and flows and that these changes are part of the “life” of ECH. Consequently, there is a need for dynamic management within ECH, including the capacity for managers to intervene at specific moments to bolster social activities and/or networks, particularly during periods when the resident mix has changed. Our findings also suggest that there is a need for managers to ensure that new residents, as well as existing residents, have a “realistic picture of the diversity of need that is being catered for and periodically reminding existing residents of that fact” (West et al., 2017, p. 1889).

### Methodological considerations

While this study demonstrates the value that LQR can bring to research in social care settings, we did encounter some challenges. For example, at the specialist dementia setting, not only did we struggle to recruit sufficient residents who were able to communicate their consent to take part in the research but, over the course of the interviews, three residents were withdrawn from the study due to an inability to communicate that they wanted to take part, two died and one chose to withdraw. In addition, the quality of qualitative data collected in interviews with the remaining participants in this setting diminished over successive rounds, leading the interviewers to adopt a more informal style of interview, focusing on key questions as a means to enhance engagement while reducing any potential burden. These challenges do not negate the importance of using LQR with people who have dementia but, rather, suggest the need for reflexivity, flexibility, adequate resources and innovation on the part of researchers (McKeown, Clarke, Ingleton, & Repper, 2010) as well as the need for continuous engagement with managers, care workers and/or families to establish participants’ usual level of wellbeing and, in turn, their basis for consent (Dewing, 2008).

As in previous studies, the ongoing relationship built up between participants and individual researchers during a longitudinal study presented some ethical challenges (Calman et al., 2013). Building rapport is key to any research encounter but, in LQR particularly, there is a fine balance between building a sufficiently trusting relationship that supports repeat in-depth interviews and participants mistaking the research encounter for ongoing friendship. In this study, we kept in contact with participants between interviews by sending a thank you card and/or a Christmas card to each participant after each round and kept in email contact with each scheme, but we did not make contact with participants in any other way. Additionally, the ending of LQR has to be negotiated sensitively. In our final interviews, we reminded participants that this was the last time that we would be speaking to them individually, although we would return to the scheme to tell them what we found out.

Finally, Thomson reminds us of the significance of “perspective” in our analysis of longitudinal data and “the lack of analytic closure and the significance of the position in time and space from where the interpretation of a particular case is made” (Thomson, 2007, p. 572). This is an important reminder that, in our efforts to fully understand ECH, we must be reflexive about how our participants’ accounts and, likewise, our own interpretations of them are situated in a particular context. This is, perhaps, more pertinent given that this piece of LQR took place during a period of relentless economic and political pressure on adult social care and an increasing demand on health and care services. While these pressures may well have affected the experiences of individual residents, they also had an impact on the organizational context, particularly in those schemes that were home to a high number of publicly funded residents. Using an LQR approach allowed us to explore how these changes impacted upon the experiences and perspectives of residents over time.

## Conclusion

This paper used data collected across four rounds of interviews with older people living in ECH to illustrate the benefits of using a LQR approach to understand residents’ own experiences of their changing care needs, as well as their experiences of changes within the schemes in which they lived. The approach was not without difficulty, particularly in respect of its use with people living with dementia, as well as in terms of the management of boundaries in the relationships built up with individual participants. However, despite these challenges, this paper demonstrates the detailed nature of the longitudinal data which we collected: data that supports a more nuanced understanding of the experiences of residents living in ECH. Using LQR techniques has supported the emergence of a more dynamic picture of ECH which complements and reinforces existing literature in this field.
